# Tumor-Associated Mast Cells in Thyroid Cancer

**DOI:** 10.1155/2015/705169

**Published:** 2015-08-26

**Authors:** Carla Visciano, Nella Prevete, Federica Liotti, Gianni Marone

**Affiliations:** ^1^Department of Molecular Medicine and Medical Biotechnology (DMMBM), University of Naples Federico II, 80131 Naples, Italy; ^2^Institute of Endocrinology and Experimental Oncology (IEOS), CNR, “G. Salvatore”, 80131 Naples, Italy; ^3^Department of Translational Medical Sciences (DiSMeT), University of Naples Federico II, 80131 Naples, Italy; ^4^Center for Basic and Clinical Immunologic Research (CISI), University of Naples Federico II, 80131 Naples, Italy

## Abstract

There is compelling evidence that the tumor microenvironment plays a major role in mediating aggressive features of cancer cells, including invasive capacity and resistance to conventional and novel therapies. Among the different cell populations that infiltrate cancer stroma, mast cells (MCs) can influence several aspects of tumor biology, including tumor development and progression, angiogenesis, lymphangiogenesis, and tissue remodelling. Thyroid cancer (TC), the most frequent neoplasia of the endocrine system, is characterized by a MC infiltrate, whose density correlates with extrathyroidal extension and invasiveness. Recent evidence suggests the occurrence of epithelial-to-mesenchymal transition (EMT) and stemness in human TC. The precise role of immune cells and their mediators responsible for these features in TC remains unknown. Here, we review the relevance of MC-derived mediators (e.g., the chemokines CXCL1/GRO-*α*, CXCL10/IP-10, and CXCL8/IL-8) in the context of TC. CXCL1/GRO-*α* and CXCL10/IP-10 appear to be involved in the stimulation of cell proliferation, while CXCL8/IL-8 participates in the acquisition of TC malignant traits through its ability to induce/enhance the EMT and stem-like features of TC cells. The inhibition of chemokine signaling may offer novel therapeutic approaches for the treatment of refractory forms of TC.

## 1. Immune-Inflammatory Microenvironment and Thyroid Cancer

Thyroid cancer (TC) is the most common malignancy of the endocrine system [1]. The incidence of thyroid malignancy has been increasing over the last decade [2]. TC derived from the follicular epithelial cell can be divided into well-differentiated, poorly differentiated, and undifferentiated types on the basis of histological and clinical parameters. Differentiated thyroid carcinomas (DTCs) include papillary (PTC) and follicular (FTC) histotypes. Such tumors can be treated surgically followed by radioactive iodine therapy. Anaplastic thyroid carcinomas (ATCs) are less common, display extreme aggressiveness and early metastasis, and have a poorer prognosis. In fact, there is no effective therapy for ATC and the mean survival time is less than one year [3]. Poorly differentiated thyroid carcinomas (PDTCs) are rare and aggressive thyroid tumors that represent the bridge between DTC and ATC. Despite favourable prognosis of DTC, ~5% of them progress to radioactive iodine-refractory (RAIR) tumors, which commonly lead to death within 5 years. RAIR tumors are defined as tumors with an inability, primary or secondary to previous evidence, to uptake ^131^I of primary/metastatic tissue. Standard cytotoxic chemotherapy has limited efficacy in such cases, and this has led to the introduction of novel targeted therapies in clinical trials [4].

An association between chronic inflammation and increased susceptibility to neoplastic transformation has been documented since many years [5]. Persistent low-grade inflammation is associated with up to 20% of all tumors and modulates several tumor-promoting effects, such as proliferation and survival of malignant cells, angiogenesis and metastasis, subversion of adaptive immune responses, and altered responses to hormones and chemotherapeutic agents [6]. Inflammation may influence different stages of tumor development. In some cancer types, inflammatory conditions are present before a malignant trait occurs. In other cancer types, the establishment of an inflammatory microenvironment can be observed only after the oncogenic event has occurred.

It has been suggested that the mutual influence between inflammation and cancer takes place through intrinsic and extrinsic pathways [7]. The intrinsic pathway is enabled by the activation of various types of oncogenes and/or the inactivation of tumor-suppressor genes, by different genetic events including rearrangements, deletions, amplifications, and point mutations. Transformed cells, upon activation of oncogenic signaling pathways, produce inflammatory molecules, thereby generating an inflammatory microenvironment in tumors. In the extrinsic pathway, inflammatory or infectious conditions predispose to cancer development (i.e., colon, prostate, and pancreas). The two pathways converge, resulting in the activation of transcription factors that coordinate the production of inflammatory mediators, including cytokines and chemokines. Inflammatory mediators recruit and activate various leukocytes, resulting in a cancer-related inflammatory microenvironment being generated [7].

In TC, both cancer-related inflammation pathways occur. The intrinsic pathway is activated by the most frequent genetic alterations found in PTCs, including RET/PTC, HRAS (V12), or BRAF (V600E). In human PTC, these genetic alterations activate, in a RAS-BRAF-MAPK-dependent manner, the transcription of proinflammatory molecules such as osteopontin (OPN), VEGF-A, CCL2/MCP1, CXCL1/GRO-*α*, CXCL10/IP-10, and CXCL8/IL-8. These molecules can act autocrinously, binding their receptors on TC cell surface, thus supporting the growth and survival of cancer cells [8, 9]. Alternatively, such factors can act by recruiting and functionally regulating immune cells associated with tumor stroma, thus inducing inflammation and indirectly modulating tumor-associated immune responses. Cytokines could reinforce the inflammatory milieu by inducing chemokine production by TC cells (e.g., INF-*γ* and TNF-*α* induce CXCL10/IP-10 production in primary human PTC and ATC cells) [10–12] and by activating antiapoptotic signaling in cancer cells [13].

The extrinsic pathway is characterized by an inflammatory component, including different immune cells, present both in tumor stroma and at the invasive front of TC. As far as adaptive immune response is concerned, it has been documented that PTC is often associated with autoimmune thyroid diseases (AITD), such as Hashimoto's thyroiditis (HT) and Grave's disease (GD) [14]. In other cases, PTC is associated with a lymphocytic infiltrate not linked to AITD. The impact of lymphocytic infiltrate on human PTC is still debated and probably depends on the ratio CD4/CD8 cells. Moreover, in the context of the CD4 population, high Treg density correlates with TC aggressiveness [15]. As far as innate immune response is concerned, the presence of different inflammatory cells in TC has been documented [14]. Gogali et al. found an increased number of NK cells in PTC in comparison with normal tissues. In addition, they observed a higher infiltration of immunoregulatory NK cells in PTC with respect to goiter tissues, proposing an immunoregulatory pattern of NK cells in TC, and a cytotoxic NK pattern in goiter [15, 16].

Moreover, it has been shown that the degree of lymphocyte and immature dendritic cell infiltration correlates with a better outcome in PTC and in follicular variant of PTC (FVPTC), suggesting a possible protective role for these cell populations in this cancer type [17, 18]. PDTCs and ATCs display an increased number of tumor-associated macrophages (TAMs) compared to DTC and normal thyroid. Moreover, macrophage density in PDTC specimens correlates with their invasive features and with a dismal prognosis [19]; in addition TAMs represent the most abundant immune cell type infiltrating PDTC and ATC [19, 20].

Thus, both innate and adaptive immune responses seem to be engaged in thyroid carcinogenesis. In this review, we would like to highlight the relevance of MCs and their mediators in sustaining TC growth and progression. We will also discuss how therapeutic targeting of these cells and/or of their mediators could represent novel avenues of treatment for TC histotypes unresponsive to currently available therapies.

## 2. Mast Cells and Cancer

Mast cells (MCs) originate in the bone marrow, enter the circulation as immature precursors, and reside in virtually all vascularized tissues [21]. Once settled into a tissue, they undergo maturation, taking on characteristics specific for that tissue. The c-kit receptor ligand, Stem Cell Factor (SCF), is the most relevant factor for human MC maturation and differentiation [22]. MCs are involved in both the innate and the adaptive arms of immunity and represent versatile cells that can have effector or immunomodulatory functions. MCs are important effector cells in antigen-induced anaphylaxis and other acute IgE-dependent allergic reactions [23]. MCs can be activated by immunologic or nonimmunologic stimuli and, depending on the type of activation, release a specific profile of mediators. Cross-linking of IgE bound to Fc*ε*RI expressed on the plasma membrane of MCs induces the activation of downstream events leading to the secretion of biologically active molecules implicated in allergic reactions. Non-IgE-mediated stimuli can also activate MCs (e.g., cytokines, chemokines, and endogenous danger signals). MC activation results in the release of several proinflammatory factors including preformed mediators (histamine, tryptase, chymase, carboxypeptidase A, and proteoglycans) stored in secretory granules and* de novo* synthesized lipid mediators (cyclooxygenase-derived PGD_2_ and PGE_2_ and 5-lipoxygenase-derived LTC_4_, LTD_4_, and LTE_4_), cytokines (TNF-*α*, IL-13, IL-3, GM-CSF, and IL-5), chemokines (CXCL8/IL-8, CCL3/MIP-1*α*, CXCL1/GRO-*α*, and CXCL10/IP-10), and angiogenic/lymphangiogenic factors (VEGF-A, VEGF-B, VEGF-C, and VEGF-D) [24].

It is well established that various types of hematologic malignancies and solid cancers are associated with increased MC density. In many tumors, MCs have been associated with tumor promotion and progression. MC counts have been reported to correlate with tumor stage, prognosis, and invasiveness [22, 25–27]. Increased number of MCs was found in human lymphoid neoplasms such as Hodgkin's lymphoma [27], B-cell non-Hodgkin's lymphoma [28], and primary cutaneous lymphoma [29]. Similar data were obtained in human solid cancers, such as pancreatic cancer [30], prostate cancer [31], and many others [22, 26].

In a model of prostate cancer, the TRAMP mice (transgenic adenocarcinoma of the mouse prostate), expressing the SV40 T antigen oncoprotein under the prostate-specific rat probasin promoter, MCs increased at the onset of the disease [25]. By using TRAMP mice in a MC-deficient genetic background, it has been shown that MCs are necessary for development and progression of transplanted prostate cancer [25]. In a model of pancreatic cancer, it has been shown that MC-conditioned medium induces migration, proliferation, and invasion of pancreatic cancer cell lines. In addition, pancreatic cancer cells stimulated migration of MCs, confirming the reciprocal attraction of MCs and cancer cells and the important role of MCs in tumor invasion [30, 32]. In a murine model of pancreatic cancer, MC density was increased and correlated with poor prognosis [32].

There is evidence that MC may contribute to tumor progression by supporting angiogenesis [33]. We have demonstrated that human MCs produce a large array of angiogenic (VEGF-A and VEGF-B) and lymphangiogenic (VEGF-C and VEGF-D) molecules [24]. In addition, human MCs express VEGF receptors 1 (VEGFR-1) and 2 (VEGFR-2), the coreceptors neuropilin-1 (NRP-1) and neuropilin-2 (NRP-2), and the Tie1 and Tie2 receptors [34]. These findings indicate that human MCs might participate in the complex network involving inflammation and tumor angiogenesis and lymphangiogenesis [35]. MC infiltrate correlates with microvessel density and tumor progression in melanoma [36], non-small cell lung cancer [37], and squamous cell carcinoma of the oesophagus [38]. In cutaneous lymphoma, increased MC number and degranulation correlate with microvessel density and tumor progression [29].

In support of MC role in angiogenesis, different studies using cancer-prone mice in a MC-deficient genetic background found reduced tumor angiogenesis associated with decreased tumor growth with respect to MC-proficient mice [39]. Gounaris et al. demonstrated that polyp growth is dependent on MCs in a murine model of colon carcinogenesis. In fact, MC depletion, either pharmacologically or through reconstitution of irradiated wild-type mice with MC-deficient bone marrow, leads to remission of polyps [40]. In a tumor model of myc-induced pancreatic islet tumor, development of the neoplastic lesions was associated with recruitment of MCs. MC ablation through genetic depletion or pharmacologic approaches inhibited tumor expansion [41].

Although the majority of studies indicate a protumorigenic role for MC in tumor growth and expansion, few reports have linked MCs with tumor repression. In some cancer types, MC density is correlated with a good prognosis [42, 43]. In human colorectal carcinoma, samples with low MC number displayed more pronounced invasion than those with higher numbers of MCs and the 5-year survival rate was worse in patients with low MC counts compared to those with high MC density [42]. In breast cancer, stromal MCs were found to correlate with low-grade tumors and a favourable prognosis. Patients with axillary lymph-node metastases showed more MCs in the noninvolved axillary lymph nodes, suggesting a protective effect of MCs in breast cancer [43].

Taken together these reports suggest either beneficial or detrimental roles for MCs in different cancer types, or alternatively different functions in cancer development, depending on the stage of the particular tumors.

## 3. Mast Cells in Thyroid Cancer

The relationship between MCs and thyroid cancer (TC) was investigated for the first time by our group [26]. Normal thyroid tissues stained essentially negative for tryptase, a specific MC marker, whereas in 95% of PTC samples we found a MC infiltration whose extent correlated with extrathyroidal extension of tumors. We also interrogated, by IHC, a limited number of PDTCs and ATCs for MC presence. We found that MCs are present in PDTC and ATC, and their density correlates with tumor invasiveness [44]. Taken together these preliminary data indicate that MCs play a role in aggressive TC. Proietti et al. evaluated the presence and distribution of MCs in follicular variant of PTC (FVPTC) and follicular adenoma. MCs were significantly more abundant in the intratumoral and peritumoral areas of FVPTC compared to adenoma [18]. Thus, MC density could be used to distinguish between benign and malignant forms of follicular thyroid lesions [18].

*In vitro* studies, conducted by using different human mast cell lines (HMC-1 and LAD2) and human primary MC derived from lung (HLMC), revealed that VEGF-A, produced by various TC cell lines, induced MC chemotaxis. Moreover, other soluble factors released by TC cells caused MC activation. This activation was non-IgE-mediated, and the TC-derived mediators that induce such phenomenon are still unknown. By analysing MC factors released upon TC activation, we identified different mediators, including histamine, IL-1, TNF-*α*, IL-6, and the chemokines CXCL1/GRO-*α*, CXCL10/IP-10, and CXCL8/IL-8. Mediators present in MC-conditioned media (MC CM) stimulated TC cell proliferation, survival, and motility. Histamine, by binding to H_1_ and H_2_ receptors expressed on PTC cells, induced an enhancement of cell proliferation. The effect exerted by histamine on TC cell proliferation was lower than that induced by MC CM. The combination of histamine with CXCL1/GRO-*α* and CXCL10/IP-10 induced TC cell proliferation with an efficiency comparable to that of MC CM. Immune-depletion experiments confirmed that histamine and the chemokines CXCL1/GRO-*α* and CXCL10/IP-10 were the main factors responsible for MC CM-dependent cell proliferation of TC cells [26]. Subcutaneous coinjection of MCs and TC cells accelerated the growth of TC xenografts in athymic mice. TC cell xenografts recruited MCs injected into the tail vein in tumor site. In addition, MC injection promoted increased proliferation and vascularization of xenografts. These effects were inhibited by treatment of mice with sodium cromoglycate (cromolyn), a specific inhibitor of MC degranulation [26]. Collectively, these results support the hypothesis that MCs and their mediators play a protumorigenic role in TC.

## 4. Mast Cells, Epithelial-to-Mesenchymal Transition (EMT), and Stemness

Our previous results indicate that MCs are present in PTC and that their density correlates with an invasive behaviour [26]. The increased motility and invasiveness of tumor cells are a consequence of the epithelial-to-mesenchymal transition (EMT) activation; this is an important step in tumor progression, responsible for the acquisition of invasive properties by tumor cells [45]. EMT is a well characterized genetic program, through which epithelial cells transdifferentiate and acquire a mesenchymal and invasive phenotype. EMT physiologically occurs during embryonic development or as a response to injury. At the cellular level, pathological EMTs are very similar to physiological EMTs as they are governed by similar signaling pathways, regulators, and effectors. EMT commonly occurs at the invasive front (tumor-stromal boundary) of many carcinomas [46] and can be triggered by cellular signals arising both from cancer cells and from tumor microenvironment. For this reason we investigated MC contribution to EMT of TC cells.

Human TC cell lines, derived from PTC, FTC, and ATC, when exposed to activated MC-conditioned medium (MC CM), undergo EMT. TC cells treated with MC CM displayed morphological changes towards mesenchymal phenotype, upregulation of EMT markers, downregulation of epithelial markers, and the activation of a functional EMT [44].

MCs can produce a wide spectrum of different mediators, both in resting conditions and upon activation (IL-6, CXCL8/IL-8, CCL25/TECK, CXCL10/IP-10, CXCL1/GRO-*α*, and TNF-*α*). Among these mediators, TNF-*α*, IL-6, and CXCL8/IL-8 efficiently induced a functional EMT. However, CXCL8/IL-8 but not IL-6 or TNF-*α* immune-depletion ablated MC CM-mediated EMT induction in TC cells. This effect was reverted by adding exogenous CXCL8/IL-8. These data suggest that CXCL8/IL-8 is required for EMT [44].

The relationship between EMT and cancer stem cells (CSCs) has been documented in different cancer types [47]. EMT inducers or regulators could also induce cancer cells to acquire stem cell-like characteristics, indicating that there is a crosstalk between the EMT program and the pathways involved in the regulation of stemness [47]. A way to isolate cells with stem-like features is to exploit their ability to grow in low adherence conditions, which favour the formation of cell spheroids [48–50]. MC CM or recombinant CXCL8/IL-8 treatment of TC cells caused the acquisition of stemness features with higher efficiency compared to unstimulated cells, demonstrating that MC CM and CXCL8/IL-8 induce/potentiate TC cell stemness. The blockade of CXCL8/IL-8 receptors CXCR1 and CXCR2 with neutralizing antibodies caused a marked reduction in sphere-forming ability of TC cells [44]. We found that CXCL8/IL-8 stimulation induced/enhanced EMT/stemness of TC cells via an Akt-SLUG pathway [44]. Human PTC samples, analyzed by immunohistochemistry with antibodies anti-tryptase and anti-OCT-4, a stem cell marker, exhibited various degrees of stained cells. A positive correlation between MC density (tryptase positive cells) and stemness features (OCT-4) was found. Moreover, each of these parameters significantly associated with a higher T [44]. Taken together, these results indicate that MCs, by releasing specific mediators, including CXCL8/IL-8, enhance the acquisition of mesenchymal and stem-like features of TC cells, thus promoting cancer progression.

## 5. Closing Thoughts

The recognition that tumor initiation and progression are greatly influenced by tumor cell interaction with stroma has led to the notion that cancer management may be improved by therapeutic targeting of the tumor microenvironment [51]. TC represents an example of such concept. TC is infiltrated and surrounded by various immune cells, whose role is still not completely understood. For instance, it has been demonstrated that tumor-associated macrophages play a tumorigenic role in TC [19, 20, 52].

We have recently suggested a protumorigenic role for MCs in TC [26]. DTC is frequently associated with AITD, and some authors suggest that AITD may predispose to TC. Interestingly, the number of MCs in thyroid was significantly higher in animals prone to develop spontaneous autoimmune thyroiditis than in control animals, suggesting that MCs may play a role in this disease [53]. Moreover, MCs were found at tissue-regenerative sites in human samples of subacute thyroiditis, suggesting a role in the thyroid tissue repair and remodeling [54]. MC infiltration is present not only in AITD, but also in human TC.

TC cells recruit and activate MCs into the tumor site; on the other hand, activated MCs release a wide spectrum of growth factors, angiogenic factors, and proinflammatory molecules responsible for TC aggressive phenotypes [26]. CXCL1/GRO-*α* and CXCL10/IP-10, together with histamine, were responsible for PTC cell proliferation. In addition, MCs and their mediators enhanced migratory and invasive ability of TC cells through the induction of EMT and stemness. Our experiments demonstrated that MC-derived CXCL8/IL-8 induced EMT and stemness by an Akt-SLUG dependent pathway [44]. SLUG is associated with high TNM stage and lymph-node metastasis in PTC [52, 55, 56] and we found that it is needed for EMT and stemness in TC, as shown by RNA interference experiments [44]. In support of these observations, we have shown that MCs and OCT-4 staining were positively correlated in human PTC samples. Thus, such data indicate that MCs, recruited and activated in TC site, produce various soluble factors with protumorigenic activity.

To definitively demonstrate whether and to what extent MCs are indeed required for thyroid carcinogenesis, we used an* in vivo* approach. To this aim we intercrossed PTC-prone mice (TG-TRK and TG-RET/PTC3 mice) with MC-deficient W^sh/sh^ mice. Our preliminary data indicate that the percentage of thyroid neoplastic lesions, caused by thyroid-restricted TG-TRK and TG-RET/PTC3 transgene expression, in the MC-deficient background was significantly lower than that observed in MC-proficient background (Liotti et al., unpublished data).

It is important to note that MC-derived mediators, important in TC, have been also described to play a role in several other cancer types. There is some evidence that histamine is involved in cell proliferation, embryonic development, and tumor growth favouring the proliferation of normal and malignant cells [57, 58]. Astemizole, a H_1_ receptor antagonist, can inhibit cell proliferation [57]. Malignant melanoma cells exhibit high constitutive levels of CXCL1/GRO-*α* that is not expressed constitutively in normal human epidermal melanocytes. Furthermore, CXCL1/GRO-*α* overexpression has been related to genesis and progression of human melanoma [59]. CXCL1/GRO-*α* mediates lung metastasis and chemoresistance in breast cancer. Consistently, blocking this axis in combination with chemotherapy reduced metastatic burden in preclinical model of breast cancer [60]. CXCL10/IP-10 exhibits tumor-promoting ability: CXCR3 and its ligands CXCL10/IP-10 may be involved in tumor progression and metastasis in human breast cancer cell lines. CXCL10/IP-10 has also been reported to promote colon cancer metastasis and to enhance tumorigenesis in basal cell carcinoma and human glioma [61]. Finally, it has been demonstrated that many types of human carcinomas (breast, colon, cervical, gastric, lung, ovarian, and medullary thyroid carcinoma) express high levels of CXCL8/IL-8 compared to normal tissues [62–64]. In addition, multiple clinical studies in medullary thyroid carcinoma, melanoma, breast, ovarian, and prostate cancer have shown a direct correlation between serum CXCL8/IL-8 levels and disease progression [65, 66]. In recent years, it has also been shown that a link exists between CXCL8/IL-8 and tumor stemness. The crucial role of CXCR1/IL-8 axis has been well documented in breast and melanoma cancer stem cells (CSCs). Populations of CSCs have been shown to express elevated levels of CXCR1 and the addition of CXCL8/IL-8 to epithelial breast cancer cells increased the number of CSCs. Blockade of receptor activity via neutralizing antibodies or through the small-molecule CXCR1 inhibitor, repertaxin, decreased the breast CSC population both* in vitro* and* in vivo* [67–69]. Additional studies conducted with primary colorectal cancer cells transfected with the stem cell associated transcription factor OCT-4 also showed that CSCs secrete high levels of CXCL8/IL-8 and that neutralizing antibodies against this molecule were able to inhibit tumor-sphere formation and the expression of the CSC markers (CD133, CD44, SOX2, SNAIL, and ABCG2) and to decrease resistance to treatment [69].

**Figure 1:**
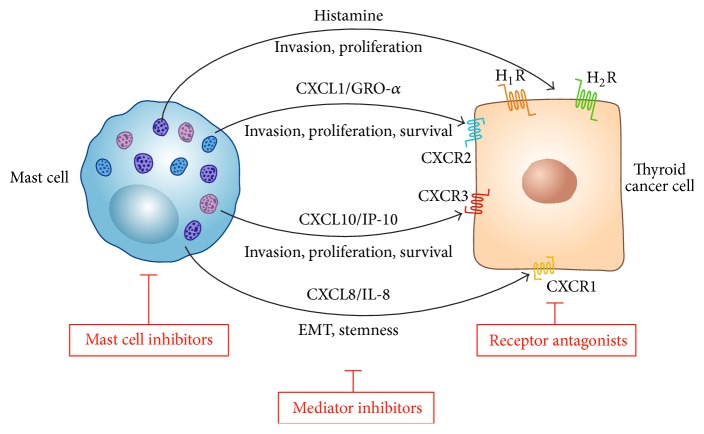
Schematic representation of the role of mast cell-derived mediators in TC progression. Activated human MCs produce a wide spectrum of preformed (e.g., histamine) and newly synthesized mediators, including several chemokines. Histamine modulates* in vitro* proliferation and invasion of TC cells through the engagement of histamine H_1_ (H_1_R) and H_2_ (H_2_R) receptors. CXCL1/GRO-*α* and CXCL10/IP-10 modulate proliferation, survival, and invasion of TC cells through the engagement of chemokine receptors CXCR2 and CXCR3, respectively. CXCL8/IL-8 modulates the epithelial-to-mesenchymal transition (EMT) and stemness of TC cells through the engagement of CXCR1. MC inhibitors, specific inhibitors of the above mentioned mediators, and receptors antagonists might offer novel therapeutic approaches for the treatment of difficult-to-cure thyroid carcinomas.

Such studies emphasize the importance of MC-derived histamine, CXCL1/GRO-*α*, CXCL10/IP-10, and CXCL8/IL-8 in TC as essential factors for tumor proliferation and invasion and for the induction and maintenance of the mesenchymal and stem-like phenotype of aggressive, metastatic TC cells (Figure 1). MC-derived mediators can be induced by different stimuli [70, 71], the nature of which is being currently investigated.

Therapeutic strategies for aggressive TC are urgently needed and may be useful to target not only the bulk of cancer cells, but also CSCs, that are probably responsible for tumor progression and recurrence. The role of CXCL8/IL-8 in sustaining CSC population in TC is of potential interest from a therapeutic point of view. It is likely that other stromal cells, including fibroblasts (CAFs) and various immune cells, may be an additional source of CXCL8/IL-8 in the context of TC. For instance, it has been recently shown that TAMs produce high level of CXCL8/IL-8 in PTC [72, 73]. Thus, targeting MCs or the IL-8/CXCR1/CXCR2 axis could be a promising strategy to target CSCs and to improve the outcome of aggressive TC (Figure 1).
